# Monoclonal Antibodies to Meningococcal Factor H Binding Protein with Overlapping Epitopes and Discordant Functional Activity

**DOI:** 10.1371/journal.pone.0034272

**Published:** 2012-03-26

**Authors:** Serena Giuntini, Peter T. Beernink, Donald C. Reason, Dan M. Granoff

**Affiliations:** Center for Immunobiology and Vaccine Development, Children's Hospital Oakland Research Institute, Oakland, California, United States of America; Wadsworth Center, New York State Dept. Health, United States of America

## Abstract

**Background:**

Meningococcal factor H binding protein (fHbp) is a promising vaccine candidate. Anti-fHbp antibodies can bind to meningococci and elicit complement-mediated bactericidal activity directly. The antibodies also can block binding of the human complement down-regulator, factor H (fH). Without bound fH, the organism would be expected to have increased susceptibility to bacteriolysis. Here we describe bactericidal activity of two anti-fHbp mAbs with overlapping epitopes in relation to their different effects on fH binding and bactericidal activity.

**Methods and Principal Findings:**

Both mAbs recognized prevalent fHbp sequence variants in variant group 1. Using yeast display and site-specific mutagenesis, binding of one of the mAbs (JAR 1, IgG3) to fHbp was eliminated by a single amino acid substitution, R204A, and was decreased by K143A but not by R204H or D142A. The JAR 1 epitope overlapped that of previously described mAb (mAb502, IgG2a) whose binding to fHbp was eliminated by R204A or R204H substitutions, and was decreased by D142A but not by K143A. Although JAR 1 and mAb502 appeared to have overlapping epitopes, only JAR 1 inhibited binding of fH to fHbp and had human complement-mediated bactericidal activity. mAb502 enhanced fH binding and lacked human complement-mediated bactericidal activity. To control for confounding effects of different mouse IgG subclasses on complement activation, we created chimeric mAbs in which the mouse mAb502 or JAR 1 paratopes were paired with human IgG1 constant regions. While both chimeric mAbs showed similar binding to fHbp, only JAR 1, which inhibited fH binding, had human complement-mediated bactericidal activity.

**Conclusions:**

The lack of human complement-mediated bactericidal activity by anti-fHbp mAb502 appeared to result from an inability to inhibit binding of fH. These results underscore the importance of inhibition of fH binding for anti-fHbp mAb bactericidal activity.

## Introduction

An effective vaccine against disease caused by capsular group B strains of *Neisseria meningitidis* is not yet available (Reviewed in [1]). The group B capsular polysaccharide, which consists of (2→8) N-acetylneuraminic acid, is present in many human glycoproteins [2], [3]. This molecular mimicry results in the group B polysaccharide being a poor immunogen [4]. Although controversial [5], [6], a vaccine that targets the group B capsule also may pose safety concerns by eliciting auto-reactive antibodies. Efforts, therefore, to develop group B meningococcal vaccines have focused largely on non-capsular antigens [1], [7].

One of the most promising non-capsular vaccine candidates is factor H binding protein (fHbp), which previously was referred to as GNA 1870 [8] or LP2086 [9]. fHbp is a surface-exposed lipoprotein that binds the human complement protein, factor H (fH). Bound fH down-regulates complement activation (particularly by the alternative pathway) and enhances the ability of the bacteria to survive in human serum [10]–[12] and cause invasive disease [13], [14]. fHbp is part of three vaccines in clinical development. One vaccine includes three recombinant proteins (five antigens), which are combined with detergent-treated outer membrane vesicles [15], [16]. The second contains two recombinant fHbp sequence variants from different variant groups [17]. The third vaccine consists of native outer membrane vesicles from mutant meningococcal strains with genetically attenuated endotoxin and over-expressed fHbp [18].

Based on amino acid sequence variability, fHbp has been divided into three variant groups by Masignani et al [19], or two sub-families, designed “A” and “B” by Fletcher et al [9]. Sub-family A includes variant groups 2 and 3, and sub-family B includes variant group 1. The amino acid identity between the two sub-families is approximately 65%. In general, anti-fHbp antibodies elicit complement-bactericidal activity only against strains with fHbp from the same sub-family as that of fHbp vaccine antigen [9].

Recently, the molecular architecture of fHbp also was reported to be modular with five variable segments, each flanked by invariant sequences [20]. Each of the variable segments is derived from one of two lineages, designated 1 or 2 (http://pubmlst.org/perl/bigsdb/bigsdb.pl?db=pubmlst_neisseria_seqdef&page=alleleQuery&locus=FHbp_peptide) (The lineages were originally referred to as alpha and beta, respectively [20]). fHbp amino acid sequence variants can contain all five variable segments derived from one lineage, or have variable segments from different lineages (natural chimeras). While ten modular groups have been recognized [21], 99% of all sequence variants identified to date can be categorized into six modular groups, designated I to VI.

Anti-fHbp mAbs can bind to meningococci and elicit complement-mediated bactericidal activity directly [22]. A number of factors have been found to influence susceptibility of different strains to anti-fHbp bacteriolysis. These included the amount of fHbp expressed by a strain [23]–[25], antigenic cross-reactivity [9], [26], and IgG antibody subclass [25]. In addition, anti-fHbp antibodies that blocked binding of fH would be expected to increase susceptibility of the organism to bacteriolysis [10], [12]. Support for this hypothesis came from recent studies of three chimeric human IgG1 mouse anti-fHbp mAbs, two of which inhibited binding of fH to fHbp, and had bactericidal activity [27]. However, the different locations on the fHbp molecule of the epitopes recognized by the three mAbs also may have contributed to the differences in bactericidal activity.

In the present study we determined the critical residues involved in the interaction between fHbp and a murine anti-fHbp mAb, designated JAR 1 [22], whose epitope had not been defined. JAR 1 is of interest because the mAb inhibited binding of fH to fHbp, and was bactericidal with human complement. Further, the JAR 1 epitope overlapped that of a previously described murine anti-fHbp mAb, mAb502 [28], which did not block fH binding to fHbp [27], [29], [30], and was bactericidal only with rabbit complement. The data illustrated how subtle differences in fine antigenic specificity that affect fH binding can drastically affect mAb protective activity.

## Results

### Anti-fHbp mAb JAR 1 is specific for fHbp sequence variants in group 1

**Figure 1:**
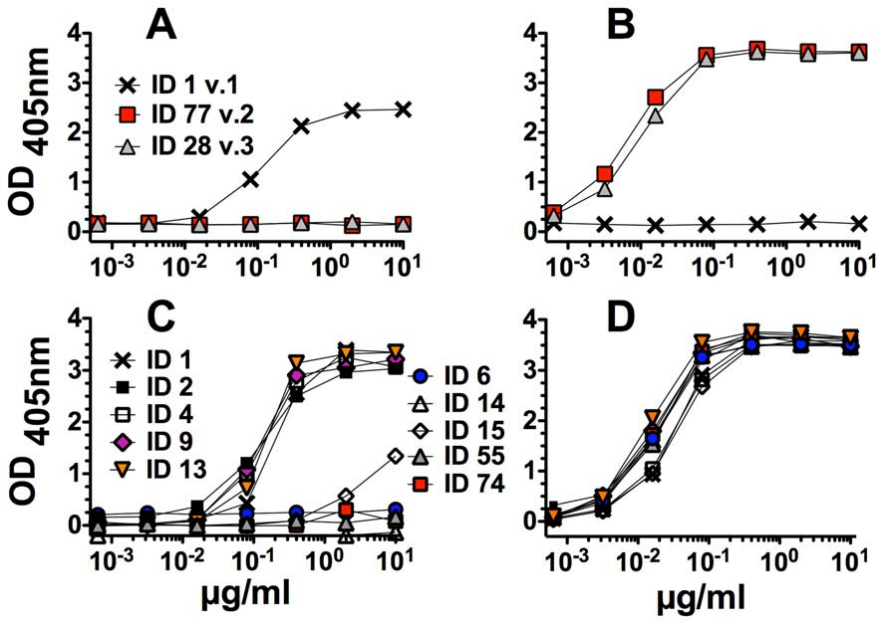
Binding of anti-fHbp mAb JAR 1 to recombinant fHbp as measured by ELISA. **Panel A.** Binding of JAR 1 to fHbp ID 1 (variant group 1); ID 77 (variant group 2); or ID 28 (variant group 3). **Panel B.** Binding of a control anti-fHbp, JAR 13, which is specific for fHbp in variant groups 2 and 3 [32]. Sequence variants tested and respective symbols are those shown in Panel A. **Panel C.** Binding of JAR 1 to variants of fHbp in variant group 1. ID 6, 14, 15, 55 and 74 are negative. ID 1, 2, 4, 9 and 13 are positive. **Panel D.** Binding of a positive control anti-fHbp mAb, JAR 65, to recombinant fHbp variants shown in Panel C. Wells of the microtiter plates were coated with 2 µg/ml of each recombinant protein.

As of January 2012, more than 500 unique fHbp amino acid sequence variants had been uploaded into the public database at http://pubmlst.org/neisseria/fHbp/. Each of these variants has been given unique identification (ID) numbers. As described in the Introduction section, fHbp sequence variants can be categorized into three variant groups [19]. We therefore tested binding of JAR 1 with eighteen recombinant fHbp sequence variants. These were selected to be representative of each of the three variant groups (Table 1), and included examples of variants expressed by prevalent disease-causing isolates [24], [31]. By ELISA, JAR 1 bound to fHbp ID 1, which is in variant group 1, but did not bind with fHbp ID 77 in variant group 2 or fHbp ID 28 in variant group 3 (Figure 1, Panel A). The presence of fHbp ID 77 or ID 22 on the plate was confirmed by binding of a second anti-fHbp mAb, JAR 13 (Panel B), which is specific for fHbp in variant groups 2 and 3 [26], [32]. In experiments not shown, JAR 1 also did not bind with two other fHbp sequence variants in variant group 2 (ID 19, 22), or four others in variant group 3 (ID 45, 67, 79, or 175).

**Table 1:** Recombinant fHbp sequence variants tested for anti-fHbp mAb reactivity.

fHbp ID*	Variant Group**	Modular Group¶	Source of Gene (Strain)	References
1	1	I	MC58	[19]
2	1	I	Synthetic†	[29]
4	1	I	M4105	[22], [24]
6	1	I	M6190	[22], [26]
9	1	I	Mali 29/07	[24], [40]
13	1	I	M982	[9], [48]
14	1	I	NZ98/254	[22], [48]
15	1	IV	NM452	[21]
55	1	VI	CDC-1573	[9]
74	1	VI	Ug 10/06	[24], [40]
19	2	VI	MD01321	[49]
22	2	III	RM1090	[26]
77	2	VI	8047	[26]
28	3	II	M1239	[26]
45	3	V	N27/00	[48]
67	3	VIII	MA-5756	[50], [51]
79	3	V	S3032	[52]
175	3	IX	synthetic††	[31]

* As designated in the fHbp public peptide database, http://pubmlst.org/neisseria/fHbp/.

**Figure 2:**
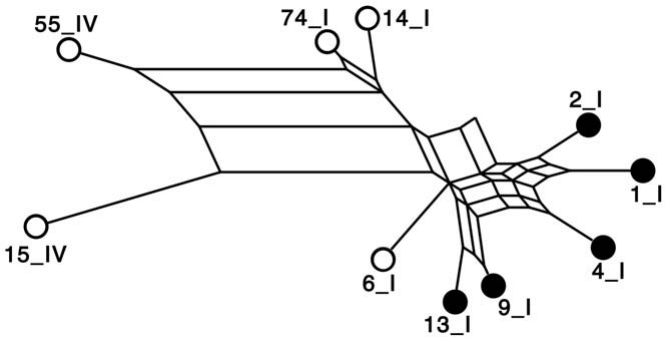
Network analysis of the relatedness of the fHbp amino acid sequence variants tested for JAR 1 binding. The network analysis was generated by the program, Splits Tree [33]. All sequence variants were in variant group 1. Each variant is designated by its peptide ID number as described in the public fHbp sequence variant database (http://pubmlst.org/neisseria/fHbp/) followed by Roman numeral I or IV, which designates the fHbp modular group [20], [21]. Sequence variants represented by filled black circles were positive for binding to JAR 1; sequence variants represented by open circles were negative for binding to JAR 1 (See Figure 1).

** Variant groups as described by Masignani et al [19]. Proteins in variant group 1 are classified as “sub-family B” by Fletcher et al [9], and proteins in variant groups 2 and 3 are classified as “sub-family A”. The relatedness of the amino acid sequences of fHbp in variant group 1 is illustrated in Figure 2.

¶ As described by Beernink and Granoff [20] and Pajon et al [21].

† codon usage was optimized for expression in *E. coli* based on the amino acid sequence obtained from the fHbp gene from strain M2197 [29].

†† codon usage was optimized for expression in *E. coli* based on the amino acid sequence obtained from the fHbp gene from strain 19498 (GenBank accession number ACI46928) [31].

We next tested binding of JAR 1 to nine other recombinant fHbp amino acid sequences in variant group 1 (Figure 1, Panel C). JAR 1 bound to ID 2, 4, 9 and 13 but not with ID 6, 14, 15, 55 or 74. The presence of each of the recombinant protein sequence variants on the plate was confirmed by binding of JAR 65 (Panel D), which is a broadly reactive anti-fHbp mAb from a mouse immunized with fHbp ID 1 (Table 1). Thus, including fHbp ID 1, JAR 1 bound to five of the 10 sequence variants tested from variant group 1.

The relatedness of each of the 10 sequence variants in group 1 was determined by a network analysis as generated by the program, SplitsTree [33] (Figure 2). The sequence variants that bound JAR 1 clustered together and were distinct from those that did not bind JAR 1. Interestingly, with one exception, the reactivity of JAR 1 with the different sequence variants paralleled that of a previously described murine IgG2a anti-fHbp mAb, mAb502 [28], [29] (data not shown). The exception was fHbp ID 2, which was positive for binding with JAR 1 but negative for binding with mAb502 (see below).

### Anti-fHbp JAR 1 recognizes an overlapping epitope with mAb502

**Figure 3:**
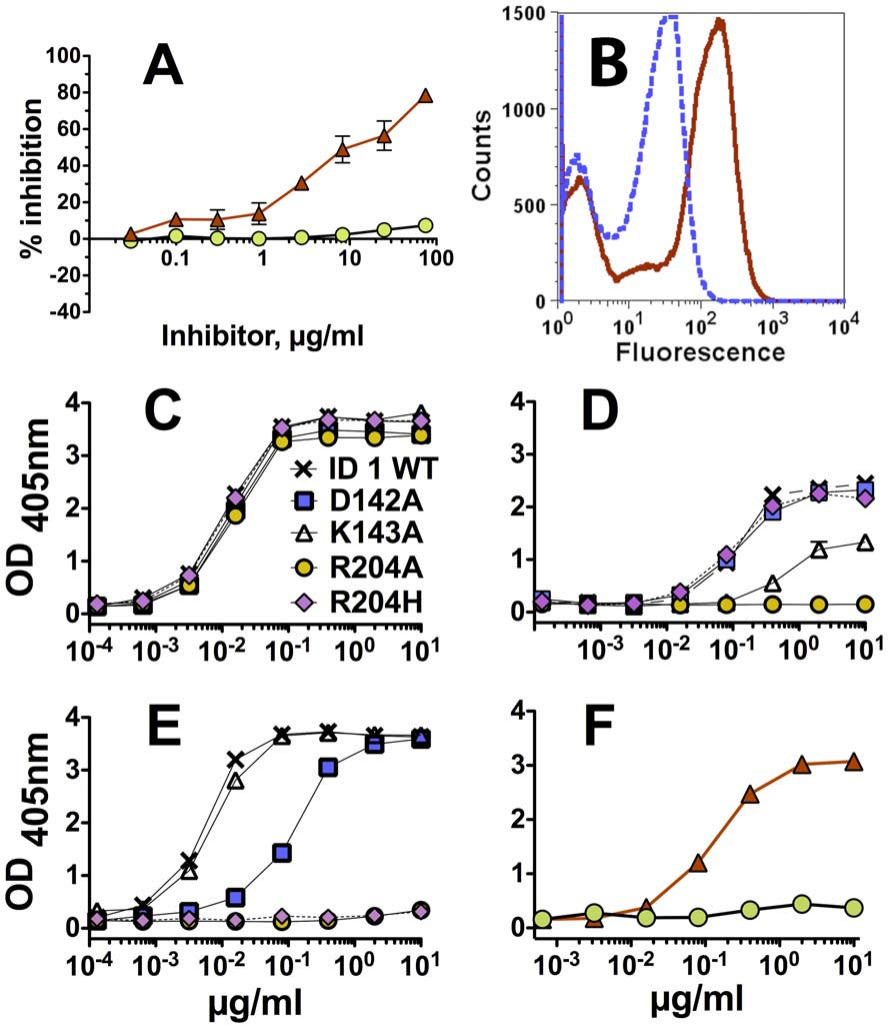
Anti-fHbp mAbs JAR 1 and mAb502 recognize overlapping epitopes on the C-terminal domain of fHb. **Panel A.** Inhibition of binding of a fixed concentration (1 µg/ml) of anti-fHbp mAbs to recombinant fHbp as measured by ELISA. Red triangles: inhibition of binding of mAb502 (IgG2a) by JAR 1 (IgG3). Green circles: Inhibition of binding of JAR 1 by mAb502. Error bars represent ranges in independent assays. **Panel B.** Binding of JAR 1 to yeast clones displaying randomly mutated fHbp on their surface. A representative clone is positive for binding to JAR 1 (red solid line) and a control anti-fHbp mAb, JAR 5 (blue dashed line). **Panels C, D, E.** Binding of anti-fHbp mAbs to mutants of recombinant fHbp as measured by ELISA. Crosses, wild-type (WT) fHbp ID 1; Blue squares, D142A mutant; White triangles, K143A mutant; Orange circles, R204A mutant; Pink diamonds with dashed black line, R204H mutant. **Panel C.** Binding of JAR 5 (positive control, data are superimposed). **Panel D.** Binding of JAR 1. **Panel E.** Binding of mAb502. **Panel F.** Binding of JAR 1 (red triangles with solid red line) or mAb502 (green circles with dashed line) to fHbp ID 2, which has H204 instead of R204 in ID 1. Wells were coated with 2 µg/ml of wild-type or mutant fHbp.

To investigate the possibility that JAR 1 and mAb502 had overlapping epitopes, we measured the ability of each of the mAbs to inhibit binding of the other to fHbp using a competitive inhibition ELISA with fHbp ID 1 as the antigen adsorbed to the wells. While JAR 1 inhibited binding of mAb502 to fHbp, mAb502 did not inhibit binding of JAR 1 (Figure 3, Panel A).

To identify amino acid residues essential for JAR 1 binding, we constructed a yeast library displaying randomly mutated fHbp ID 1 on the cell surface. Figure 3, Panel B shows an example of yeast cells that were bound by both JAR 1 and a second positive control anti-fHbp mAb JAR 5 [22], [32], as measured by flow cytometry. Yeast clones displaying fHbp mutants that were positive for JAR 5 binding but negative for JAR 1 binding were isolated by cell sorting, expanded, and retested for binding. We determined the DNA sequences encoding the mutant fHbp of 55 JAR 5-positive and JAR 1-negative clones. Representative translated amino acid sequences are shown in Figure S1. Based on the alignments, we identified clones in which single amino acid mutations eliminated JAR 1 binding. These residues included Asp at position 142 (D142), Lys at position 143 (K143), and Arg at position 204 (R204).

To determine whether the residues identified by the yeast display studies affected JAR 1 epitope expression, we used site-directed mutagenesis to replace each of the individual residues by alanine, and tested the mutant recombinant proteins for binding with JAR 1 by ELISA. All three mutants, and a fourth mutant, R204H (see below) showed similar concentration-depending binding with the positive control anti-fHbp mAb, JAR 5 (Figure 3, Panel C). For JAR 1 (Panel D), the D142A mutation (blue squares) had no effect on binding, the K143A mutation (open triangles) partially decreased binding, and the R204A mutation (orange circles) resulted in complete loss of binding. We also prepared a R204H mutant since we had shown that JAR 1 bound to the natural fHbp sequence amino acid variant ID 2 (Figure 1, Panel C), which has His at position 204 instead of Arg. Although substitution of Ala for Arg at residue 204 eliminated binding of JAR 1 to fHbp ID 1 (R204A), substitution of His for Arg at residue 204 of ID 1 did not affect JAR 1 binding (R204H, Figure 3, Panel D).

In a previous study, mAb502 was shown to bind to an epitope located in the C-terminal domain involving Arg204 [28], [29]. In our studies, both the R204A (orange circles, solid line) and R204H mutations (magenta diamonds, dashed lines) in fHbp ID 1 eliminated mAb502 binding (Figure 3, Panel E). The lack of binding of mAb502 to the R204H mutant was consistent with lack of binding of this mAb to wild-type fHbp ID 2 that contains His at residue 204 (green circles, Figure 3, Panel F). The D142A mutation in fHbp ID 1, which had no effect on binding of JAR 1, decreased binding of mAb502 (Panel E). Conversely, the K143A mutation, which partially decreased binding of JAR 1, had no effect on binding of mAb502. These results, together with the data that JAR 1 inhibited binding of mAb502 to fHbp, indicated that the two anti-fHbp mAbs recognized overlapping but distinct epitopes.

### JAR 1 and mAb502 differ in their ability to inhibit binding of fH, and activate complement-mediated bacteriolysis

**Figure 4:**
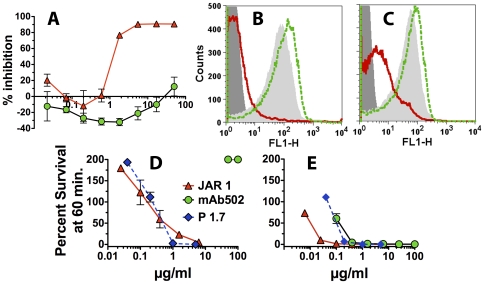
Inhibition of binding of fH in relation to anti-fHbp mAb bactericidal activity. **Panel A.** Inhibition of fH binding to recombinant fHbp as measured by ELISA. A fixed concentration of human fH (2 µg/ml) was incubated with different concentrations of anti-fHbp mAb JAR 1 (red triangles with solid red line) or mAb502 (green circles with dashed green line). **Panels B.** Inhibition of fH binding to live bacteria of wild-type group B strain H44/76 as measured by flow cytometry. A fixed concentration of fH (∼90 µg/ml) was incubated with 2 µg/ml of JAR 1, or 2 or 50 µg/ml of mAb502. The source of human fH was 20% IgG-depleted human serum. Dark gray filled area, bacteria without fH as a negative control; Light gray filled area, fH without the addition of a mAb; fH and JAR 1 (red line); fH and mAb502, (green dashed line). Data for 2 or 50 µg/ml of mAb502 were similar; only 50 µg/ml histogram is shown. **Panel C.** Inhibition of fH binding to live bacteria of a mutant of group A strain Senegal 1/99 with over-expressed fHbp ID 5 as measured by flow cytometry. Symbols same as in Panel B. **Panel D.** Survival of *N. meningitidis* group B H44/76 strains after 60 min incubation with anti-fHbp mAbs and 20% human complement. JAR 1 (red triangles) and the anti-PorA P1.7 mAb control were bactericidal but not mAb502 (green circles). **Panel E.** Same as Panel D except infant rabbit complement was used instead of human complement. All three mAbs had activity. For each panel, the respective results were replicated in two independent experiments. Error bars represent ranges in values.

By ELISA, JAR 1 inhibited binding of fH to fHbp while mAb502 did not inhibit fH binding. Indeed, mAb502 enhanced fH binding to fHbp (Figure 4, Panel A). By flow cytometry, mAb502 also did not inhibit binding of fH to the bacterial surfaces of group B strain H44/76 (Panel B), or a mutant of group A strain Senegal 1/99 with increased expression of fHbp ID 5 (Panel C). For both strains there appeared to be a small but reproducible increase in fH binding in the presence of mAb502. In contrast, JAR 1 inhibited fH binding to both strains. The lower JAR 1 inhibition of fH binding with the group A strain likely reflected fH bound by a second ligand, NspA [34], which has higher expression in this test strain than in the group B H44/76 test strain.

In a bactericidal assay with human complement (Figure 4, Panel D), JAR 1 and a positive control murine anti-PorA P1.7 mAb, killed group B strain H44/76 while mAb502, which did not inhibit fH binding, had no activity (200% survival of the bacteria after 1 hr incubation, which reflected an increase in the CFU compared to time 0). In contrast, with infant rabbit complement, all three mAbs had bactericidal activity (Figure 4, Panel E). Binding of fH to *N. meningitidis* is specific for human fH [13]. Thus in the presence of rabbit fH, which did not bind to the bacteria, mAb502 was bactericidal.

### Chimeric human IgG1 JAR 1 but not mAb502, is bactericidal with human complement

The four mouse IgG subclasses have different complement activation properties [35]. In order to investigate whether or not the greater bactericidal activity of JAR 1 (IgG3) than mAb502 (IgG2a) resulted from different subclasses, we sequenced the variable region genes encoding JAR 1 and created a chimeric human IgG1 mouse JAR 1 mAb (See Methods). The activity of the chimeric JAR 1 was compared to that of chimeric human IgG1 mAb502, which we had prepared in a previous study [27].

**Figure 5:**
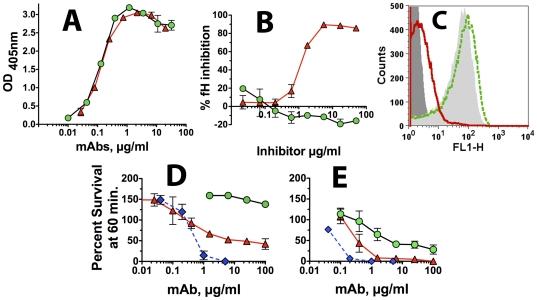
Activity of chimeric human IgG1 mouse anti-fHbp mAbs. **Panel A.** Binding of chimeric mAbs to fHbp by ELISA using anti-human kappa light chain secondary antibody (See Methods). Red triangles with solid red line, chimeric JAR 1; Green circles with dashed line, chimeric mAb502. **Panel B.** Inhibition of fH binding to recombinant fHbp as measured by ELISA. Human fH (2 µg/ml) was incubated with different concentrations of chimeric JAR 1 or chimeric mAb502. Symbols as in Panel A. **Panel C.** Inhibition of binding of fH to live bacteria of group B strain H44/76 as measured by flow cytometry. A fixed concentration of human fH (∼90 µg/ml) was incubated with 2 µg/ml of chimeric JAR 1, or 2 or 50 µg/ml of chimeric mAb502. The source of fH was 20% IgG-depleted human serum. Dark gray filled area, bacteria without fH as a negative control; Light gray filled area, fH without the addition of a mAb; fH and JAR 1 (solid red line); fH and mAb502 (green dashed line). Data for 2 or 50 µg/ml of chimeric mAb502 were similar; only the 50 µg/ml histogram is shown. **Panel D.** Survival of wild-type group B strain H44/76 after 60 min incubation with chimeric anti-fHbp mAbs and 20% human complement. Symbols for chimeric anti-fHbp mAbs are same as in Panel A; Blue diamonds with dashed blue line, positive control anti-PorA P1.7 mAb. Data are from two experiments. Error bars, range in values. **Panel E.** Same as Panel D except that infant rabbit complement was used instead of human complement.

In an ELISA with recombinant fHbp ID 1 adsorbed to the wells of a microtiter plate, the two human IgG1 chimeric mAbs showed similar respective binding (Figure 5, Panel A). As observed with the corresponding mouse mAbs, the chimeric JAR 1 mAb inhibited binding of fH to fHbp, while the chimeric mAb502 mAb gave slightly more fH binding (seen as negative inhibition, Figure 5, Panel B). By flow cytometry, there also was slight enhancement of fH binding to live bacterial cells of group B strain H44/76 by the chimeric mAb502 (Figure 5, Panel C). In contrast, as little as 2 µg/ml of the chimeric JAR 1, inhibited binding of fH to the surface.

With human complement, the chimeric human IgG1 JAR 1 mAb (red triangles) had bactericidal activity against strain H44/76 while the chimeric IgG1 mAb502 (green circles) was not bactericidal (Figure 5, Panel D). The mouse anti-PorA P1.7 mAb (blue diamonds, dashed lines) had the strongest activity. With rabbit complement, both anti-fHbp mAbs, and the control mouse anti-PorA mAb, had bactericidal activity (Figure 5, Panel E).

## Discussion

fHbp consists of two domains of antiparallel β-strands connected by a five amino acid linker [36]–[38]. In previous studies, our laboratory produced a panel of anti-fHbp mAbs from mice immunized with fHbp sequence variants from each of the three variant groups [22], [32], [39]. The majority of the mAbs recognized epitopes located in either a conserved portion of the N-terminal domain, or more variable regions in the C-terminal domain of the protein [32], [39]. In the present study, we have characterized the functional properties of anti-fHbp mAb, JAR 1, and the amino acid residues affecting the JAR 1 epitope, which had not been defined. The results were compared to those of a previously described anti-fHbp mAb, mAb502 [28], [29].

Both JAR 1 and mAb502 bound to a subset of fHbp sequences in variant group 1, which included ID 1, 4 and 9. In previous studies, fHbp ID 1 was expressed by prevalent group B strains responsible for meningococcal disease in the United States and Europe [31]; and fHbp ID 9 was expressed by W-135 isolates causing epidemic meningococcal disease in Africa [24], [40]. Interestingly, fHbp ID 4 is associated both with group B strains causing meningococcal disease in the UK [31], and group A isolates causing epidemics in the Africa [24], [40].

Based on previous studies, the epitope recognized by mAb502 was known to be located on the C-terminal domain of fHbp [29]. The mAb was bactericidal with rabbit complement [28], and did not inhibit binding of human fH [29]. Data from the present study suggested that JAR 1 and mAb502 recognized overlapping epitopes. Thus, by ELISA, an excess of JAR 1 inhibited >90% of binding of mAb502 to fHbp (Figure 3, Panel A); for reasons that were unclear, however, the converse was not true (an excess of mAb502 did not inhibit binding of JAR 1). The lack of mAb502 inhibition of JAR 1 binding did not appear to have resulted from low mAb502 binding avidity since in a previous study mAb502 had high avidity measured by surface plasmon resonance (a rapid association rate (*k_a_*) to immobilized fHbp, and a dissociation rate that was too slow that the actual avidity constant couldn't be calculated [27]).

**Figure 6:**
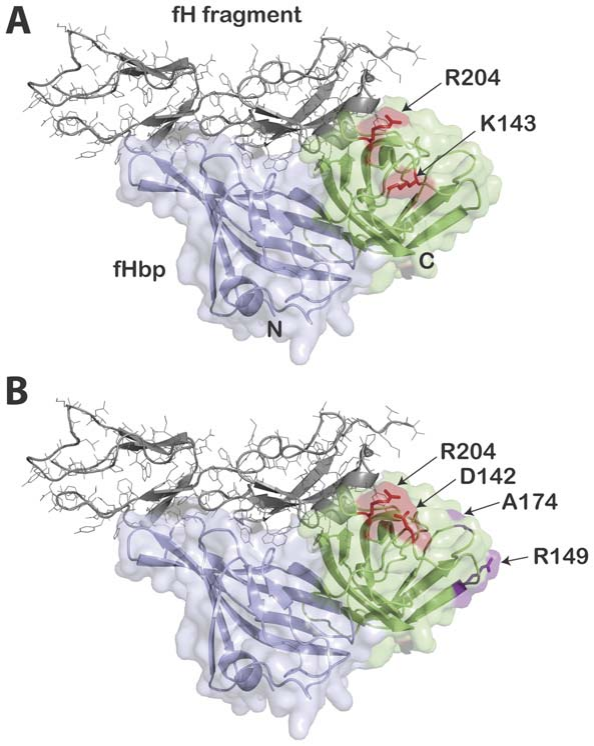
Structural model of fH-fHbp complex. fH fragment is shown in gray. The amino-terminal domain of fHbp is shown in light blue, while the carboxy-terminal domain is shown in light green. **Panel A.** Alanine substitutions for arginine at residue 204 (R204A) or for lysine at position 143 (K143A) decreased or eliminated JAR 1 binding. His substitution for Arg at position 204 (R204H) did not affect JAR 1 binding (See Figure 5). **Panel B.** Residues shown previously by NMR to be part of the mAb502 binding site [29]. In addition, we found that alanine or histidine substitutions for arginine at residue 204 (R204A or R204H) eliminated binding of mAb502, and an alanine substitution for aspartic acid at residue 142 (D142A) decreased binding (See Figure 3). The figure was constructed with PyMol (http://www.pymol.org).

Binding of both JAR 1 and mAb502 to recombinant mutants of fHbp was eliminated by a single amino acid substitution (Ala for Arg at residue 204). While mAb502 binding also was eliminated by substituting His for Arg204, this substitution did not affect JAR 1 binding. Binding of JAR 1, but not mAb502, to the natural fHbp sequence variant ID 2, which has His at residue 204, was consistent with the respective data with binding to the R204H mutant from fHbp ID 1. Thus, Arg at position 204 appears to be necessary for binding of mAb502, whereas another positively charged residue, His, at this position is sufficient for binding of JAR 1. The effects of these substitutions at 204 are likely to be direct effects given the spatial proximity of other residues affecting binding of the two mAbs (see below). Binding of JAR 1 also was decreased by the K143A substitution but not by the D142A substitution; the reverse was observed with mAb502 (binding was decreased by the D142A substitution but not by the K143A substitution). On a structural model, which shows the portion of the fHbp molecule in contact with a fragment of fH, the Asp142 and Lys143 residues on fHbp are located in proximity to Arg204 on the C-terminal domain of fHbp (D142, K143 and R204, respectively, Figure 6, Panels A and B). Interestingly, none of these amino acid substitutions affected binding of fH to fHbp (Figure S2). Small differences between the locations of the respective epitopes recognized by JAR 1 and mAb502 in relation to the fHbp residues known from the crystal structure to be in contact with fH, and/or their respective orientations of mAb binding, may explain the ability of JAR 1 but not mAb502 to inhibit fH binding. Alternatively, binding of the mAbs may have induced different conformational changes in fHbp, which in the case of JAR 1 decreased fH binding, and with mAb502 enhanced fH binding. Distinguishing between these possibilities will require additional study.

In a previous study, we showed that inhibition of fH binding was essential for bactericidal activity of human IgG1 chimeric mouse anti-fHbp mAbs [27]. Our conclusions, however, were based on data from only two chimeric anti-fHbp mAbs that blocked fH binding (JAR 3 and JAR 5) and chimeric mAb502, which did not block fH binding. The fHbp epitopes recognized by JAR 3 and JAR 5 required Gly121 and Lys122 while that of mAb502 required Arg204. Thus, the different locations of the JAR 3 and JAR 5 epitopes and that of mAb502 confounded the analysis of the role of fH inhibition on human IgG1 anti-fHbp mAb bactericidal activity. In the present study, JAR 1 was shown to recognize an epitope that overlapped that of mAb502. Since JAR 1 inhibited fH binding, and mAb502 did not inhibit fH binding, it was possible to investigate the role of inhibition of fH binding on human IgG1 mAb bactericidal activity independent of a large difference in the locations of the two epitopes. The chimeric IgG1 JAR 1 elicited human complement-mediated bactericidal activity against the wild-type group B strain H44/76, while the chimeric IgG1 mAb502 was not bactericidal (Figure 5, Panel D). These results were consistent with the importance of inhibition of binding of fH for eliciting human IgG1 anti-fHbp mAb bactericidal activity [27]. In our studies, binding of the mouse or chimeric human mAb502 was shown to enhance binding of fH slightly to both of the meningococcal test strains. Defining the mechanism for fH enhancement, and the role of enhanced fH binding as opposed simple lack of inhibition of fH binding, on resistance of *N. meningitidis* to anti-fHbp mAb human complement-mediated killing will require additional study.

The lack of cross-reactivity of most anti-fHbp mAbs against strains expressing heterologous fHbp variants highlights an important limitation of fHbp as a vaccine antigen, namely that natural fHbp variants elicit little cross-protective antibody responses against strains with fHbp from different sub-families or variant groups [19], [24], [41]. Information on the locations of the fHbp epitopes important for eliciting bactericidal antibodies have enabled new vaccine strategies to engineer chimeric fHbps capable of eliciting cross-reactive bactericidal antibodies against strains expressing fHbp variants from different antigen groups [26], [42]. The identification of regions of the fHbp molecule responsible for eliciting antibodies such as JAR 1, which block fH binding and activate human complement bactericidal activity, and ultimately understanding the basis for the opposite effects of mAb502, which recognizes an overlapping epitope, may aid further in the design of optimal chimeric fHbp antigens.

## Methods

### Ethics statement

Anti-fHbp mAb, JAR 1, was prepared in a CD-1 mouse in strict accordance with the recommendations in the Guide for the Care and Use of Laboratory Animals of the National Institutes of Health. The protocol was approved by the Children's Hospital & Research Center Oakland Institutional Animal Care and Use Committee. The human complement source for measuring serum bactericidal activity was serum from an adult who participated in a protocol that was approved by the Children's Hospital Oakland Institutional Review Board (IRB). Written informed consent was obtained from the subject.

### Murine mAbs

The murine fHbp-specific monoclonal antibodies (mAbs) used in this study are summarized in Table 2. All but one of the mAbs have been previously described [22], [28], [29], [32], [39]. The exception, JAR 65, was from a human fH transgenic BALB/c mouse immunized with a mutant of recombinant fHbp ID 1 in which Arg at position 41 was replaced by Ser (R41S), which eliminated fH binding [43]. All of the mAbs listed in Table 1 elicited cooperative human complement-mediated bactericidal activity in combination with certain second anti-fHbp mAbs. In addition JAR 1, 5, 13, and 65 inhibited binding of fH to fHbp; mAb502 did not inhibit fH binding. A control IgG2a anti-PorA P1.7 mAb was obtained from the National Institute for Biological Standards and Control, Potters Bar, United Kingdom (NIBSC code 01/514).

**Table 2:** Properties of the murine anti-fHbp mAbs.

mAb Designation	Recombinant fHbp Immunogen	IgG Subclass	fHbp Epitope Amino Acids*	References	Genbank accession numbers (V_L_ and V_H_ genes, respectively)
JAR 1	ID 1	IgG3	Arg204	Welsh et al [22]	JQ085280; JQ085281
mAb502	ID 1	IgG2a	Arg204**	Giuliani et al, [28] and Scarselli et al [29]	EU835941; EU835942
Control mAbs					
JAR 5	ID 1	IgG2b	Gly121 and Lys122	Welsh et al [22] and Beernink et al [32]	JF715927; JF715926
JAR 13	ID 77	IgG2a	Ser216	Beernink et al [32]	Not done
JAR 65	Mutant ID 1 with R41S	IgG1	Unknown	Unpublished	Not done

All but one of the hybridomas were from CD-1 mice. The exception, JAR 65, was from a human fH transgenic BALB/c mouse [43].

* fHbp mutants with amino acid substitutions at these positions lost (knocked-out) or gained (knocked-in) mAb binding [32], [39].

** The amide signals of Gly148, Arg149, and Ala174 showed chemical shifts by NMR upon binding with fHbp [29].

### Chimeric human IgG1 mouse anti-fHbp mAbs

The methods for sequencing the variable region genes, vector construction, transfection, antibody expression, and protein purification of chimeric human IgG1 mouse anti-fHbp JAR 1 and mAb502 mAbs have been previously described [27]. Transfected CHO cells were cultured in serum-free medium (Sigma Aldrich) for approximately 2 weeks. Antibody from the cell culture supernatant was concentrated and purified using HiTrap protein G columns (GE Healthcare) as described previously [27]. The concentrations of the human IgG-mouse chimeric mAbs were determined by a capture ELISA in which a goat anti-human κ chain specific antibody (Biosource) was adsorbed to the wells of a microtiter plate and used to capture the chimeric mAb. The bound mAb was detected with goat anti-human κ chain specific antibody conjugated with alkaline phosphatase (Biosource). The concentrations of the chimeric human mouse anti-fHbp mAbs were assigned by comparison with binding of a human IgG1 myeloma standard (Sigma).

### Anti-fHbp ELISA

Binding of the murine mAbs to recombinant fHbp (rfHbp) was measured by ELISA as described previously [27]. The wells of a microtiter plate (Immulon 2B; Thermo Electron Corp.) were coated with 2 µg/ml of rfHbp in PBS and incubated overnight at 4°C. After blocking, different concentrations of the mAb were added and incubated overnight at 4°C. The plates were washed and bound mAb was detected with goat anti-mouse or anti-human IgG (Fc specific)-alkaline phosphatase (Sigma; 1∶5000).

The ability of the anti-fHbp mAbs to inhibit binding of fH to fHbp also was measured by ELISA, which was performed as previously described [27]. Briefly, wells of a microtiter plate were incubated with rfHbp (2 µg/ml in PBS) overnight at 4°C. Dilutions of the mAbs were added and incubated at 37°C for 2 hrs, followed by the addition of 2 µg/ml of purified human fH (Complement Technology), which was incubated for an additional 1 hour at room temperature. Bound fH was detected by a sheep polyclonal antiserum to fH (Abcam), followed by washing and the addition of donkey anti-sheep IgG antibody (Sigma Aldrich) conjugated with alkaline phosphatase. The inhibition results were expressed as the percentage of inhibition of fH binding in the presence of an anti-fHbp mAb, compared with fH binding in the absence of the mAb.

### Competitive anti-fHbp inhibition ELISA

The ability of anti-fHbp mAb JAR 1 (IgG3) to inhibit binding of anti-fHbp mAb502 (IgG2a), or of mAb502 to inhibit binding of JAR 1, to solid-phase recombinant fHbp was performed as described elsewhere [44]. In brief, wells of a microtiter plate were coated with 2 µg/ml of rfHbp and incubated overnight at 4°C. Dilutions of the first mAb were added to the plate together with a fixed concentration (1 µg/ml) of the second mAb and incubated at 37°C for 2 hrs. The secondary antibodies for detection of JAR 1 or mAb502 were alkaline phosphatase-conjugated goat anti-mouse antiserum specific for IgG3 or IgG2a, respectively (Southern Biotech; 1∶2000).

### Flow cytometry

The ability of the mAbs to inhibit binding of fH to live bacteria was measured by flow cytometry, which was performed as described previously [27]. The group B test strain was H44/76 (B:15:P1.7,16; ST-32), which expressed fHbp ID 1 in variant group 1 [22], [23]. In some experiments, we also used a mutant of group A strain, Senegal 1/99, which expressed fHbp ID5 in variant group 1, and had been engineered to have increased expression of fHbp [24]. For measurement of inhibition of fH binding to the bacterial surface, ∼10^7^ bacterial cells/ml were incubated with 50 or 2 µg/ml of anti-fHbp mAb for 30 min at room temperature, followed by the addition of ∼90 µg/ml of human fH. The source of fH was heat-inactivated (30 min at 56°C) 20% human serum that had been depleted of IgG as previously described [43]. Bound fH was detected by a sheep polyclonal antiserum to fH (LifeSpan BioSciences) followed by washing and incubation with a donkey anti-sheep IgG antibody (Invitrogen) conjugated with Alexa Fluor 488.

### Serum bactericidal assay

Complement-mediated bactericidal activity was measured as previously described [39], [43] using group B strain H44/76. The bacterial cells were grown to mid-log phase in broth culture, harvested by centrifugation and washed and resuspended in buffer as described elsewhere [27]. Immediately before performing the assay, the anti-fHbp mAbs were centrifuged for 2 h at 100,000× g to remove possible aggregates. The 40 µl bactericidal reaction mixture contained 1 to 100 µg/ml of mAb, ∼300–400 CFU of bacteria and 20% complement. The complement source was either human or infant rabbit serum. The human complement was serum from a healthy adult with normal total hemolytic complement activity and no detectable serum bactericidal antibodies against the test strain. To eliminate non-bactericidal IgG antibodies that might augment or inhibit the activity of the test mAbs, the human serum was depleted of IgG using a protein G column (HiTrap Protein G, GE Life Sciences, Piscataway, NJ), which was performed as previously described [43]. The rabbit complement was serum pooled from normal rabbits 3–4 weeks old (Cedarlane Labs). Bactericidal activity (BC_50%_) was defined as the anti-fHbp mAb concentration that resulted in a 50% decrease in CFU/ml after 60-min incubation in the reaction mixture compared with CFU/ml in negative control wells at time zero.

### Epitope mapping using yeast display

Randomly mutated libraries of fHbp ID 1 were generated by error-prone PCR. MnCl_2_ concentrations were titrated, and conditions selected such that the average number of amino acid substitutions per molecule ranged from 1 to 3. Mutated fHbp ID 1 proteins were displayed on the surface of yeast as Aga2 fusion proteins as described by Boder and Wittrup [45], [46]. The vector pYD1 and the host yeast strain EBY100 were purchased from Invitrogen (Carlsbad, CA). The mutagenic PCR products were ligated into pUC18, expanded in *E. coli*, excised, and inserted into pYD1. Yeast were transformed with the plasmid library, expanded overnight at 30°C, transferred into Galactose-containing YNB medium (Yeast Nitrogen Base w/o amino acids; Difco) to induce recombinant protein expression, and incubated for 48 hrs at 20°C. Bulk yeast cultures were simultaneously stained with anti-fHbp mAb JAR 1 conjugated to DyLight 649 and a non-competing anti-fHbp mAb, JAR 5, which was conjugated to DyLight 488. The yeast cells were sorted to select clones that had lost the ability to bind JAR 1 but retained binding to JAR 5, which verified surface expression of the fHbp construct. Single clones were re-grown, their binding profiles verified, and the sequence of the fHbp insert they contained was determined to identify amino acid substitutions that resulted in the loss of the JAR 1 epitope. A total of 55 JAR1−/JAR5+ yeast clones were analyzed and the sequences of their inserts aligned to identify altered residues that led to the loss of JAR 1 binding.

### Construction of site-specific mutants

In order to determine whether the residues identified by the yeast display studies affected JAR 1 epitope expression, we used site directed mutagenesis to individually replace each of the residues by alanine. The sequences of the mutagenic oligonucleotides were: D142A_fwd, GCCAAGCTTCCCGAAGGCG; D142A_rev, AAAAGATGTATGTTCGCCCGC; K143A_fwd, GCCCTTCCCGAAGGCGG; K143A_rev, GTCAAAAGATGTATGTTCGCCCG; R204A_fwd, GCCCATGCCGTCATCAGCG; R204A_rev, TTTTCCATCCGGCTTGATATC; R204H_fwd, AGCCGGATGGAAAACACCATGCCGTCATCAG; R204H_rev, CTGATGACGGCATGGTGTTTTCCATCCGGCT. The oligonucleotides were phosphorylated with T4 polynucleotide kinase (New England Biolabs) prior to PCR amplification. Mutants were constructed using the Phusion Site-Directed Mutagenesis Kit (Thermo Scientific) using the manufacturer's protocols. The mutagenesis reactions were transformed into chemically competent *E. coli* DH5α (Invitrogen) and independent mutant clones were verified by DNA sequencing.

### Purification of recombinant fHbp

The wild-type fHbp amino acid sequence variants and site-specific mutant proteins were expressed from the T7 promotor using the *E. coli* plasmid pET21b (Novagen) as described previously [19], [32]. The recombinant proteins were purified by immobilized metal ion chromatography using Ni-NTA agarose (Qiagen) as described previously. Purified fHbps were dialyzed against PBS, sterilized by filtration (Millex 0.22 µm; Millipore), and stored at 4°C prior to use. The protein concentrations were determined by UV absorbance (Nanodrop 1000) based on the extinction coefficient calculated from the amino acid sequence [47].

## Supporting Information

Figure S1 **Representative alignments of 13 amino acid sequences inferred from fHbp gene inserts encoding mature proteins of representative fHbp mutants in JAR 1-negative/JAR 5-positive yeast clones.** The sequences were selected based on those encoding 1 to 3 amino acid substitutions from that of wild-type fHbp ID 1 (gene encoding fHbp from strain MC58 [22].
(DOC)

Figure S2 **Binding of fH to mutants of fHbp ID 1 as measured by ELISA.** **Panel A**. Binding of fH. The D142A, K433A, R204 and R204H mutations, which affected binding of JAR 1 and/or mAb502, did not affect fH binding. The R41S mutant of fHbp ID (white circles), which was known not to bind fH, served as a negative control [43]. (**Panel B.** Binding of anti-fHbp mAb, JAR 5. Symbols same as in Panel A. Wells were coated with 2 µg/ml of wild-type or mutant fHbp.
(DOC)
