# Supplementary material for: Monoclonal Antibodies to Meningococcal Factor H Binding Protein with Overlapping Epitopes and Discordant Functional Activity
Source: PLoS One. 2012 Mar 26;7(3):e34272. doi: 10.1371/journal.pone.0034272 (PMC3312907; doi:10.1371/journal.pone.0034272)
Supplement: Figure S1 — Representative alignments of 13 amino acid sequences inferred from fHbp gene inserts encoding mature proteins of representative fHbp mutants in JAR 1-negative/JAR 5-positive yeast clones. The sequences were selected based on those encoding 1 to 3 amino acid substitutions from that of wild-type fHbp ID 1 (gene encoding fHbp from strain MC58 [22]. (DOC) [file pone.0034272.s001.doc]

**Figure S1.**

| fHbp ID1 wildtype | 10  |  CSSGGGGVAA | 20  |  DIGAGLADAL | 30  |  TAPLDHKDKG | 40  |  LQSLTLDQSV | 50  |  RKNEKLKLAA | 60  |  QGAEKTYGNG | 70  |  DSLNTGKLKN |
| --- | --- | --- | --- | --- | --- | --- | --- |
| yeast-15  yeast-54  yeast-49  yeast-42  yeast-57  yeast-13  yeast-27  yeast-17  yeast-06  yeast-14  yeast-45  yeast-55  yeast-48 | M...  M...  M...  M...  M...  M...  M...  V...  M...  M...  M...  M...  M... | ..........  ..........  ..........  ..........  ..........  ..........  ..........  ..........  ..........  ..........  G.........  ..........  .......... | ..........  ..........  ..........  ..........  ..L.......  ..........  ..........  ..........  ..........  ..........  ..........  ..........  .......... | ..........  ..........  ..........  ..........  ..........  ..........  ..........  ..........  ..........  ..........  ..........  ..........  .......... | ..........  ..........  ..........  ..........  ..........  ..........  ..........  ..........  .N........  ..........  ..........  ..........  .......... | ..........  ..........  ..........  ..........  ..........  ..........  ..........  ..........  ..........  ..........  ..........  ..........  .......... | ..........  ..........  ..........  ..........  ..........  ..........  ..........  ..........  ..........  ..........  ..........  ..........  .......... |

| fHbp ID1 wildtype | 80  |  DKVSRFDFIR | 90  |  QIEVDGQLIT | 100  |  LESGEFQVYK | 110  |  QSHSALTAFQ | 120  |  TEQIQDSEHS | 130  |  GKMVAKRQFR | 140  |  IGDIAGEHTS |
| --- | --- | --- | --- | --- | --- | --- | --- |
| yeast-15  yeast-54  yeast-49  yeast-42  yeast-57  yeast-13  yeast-27  yeast-17  yeast-06  yeast-14  yeast-45  yeast-55  yeast-48 | ..........  ..........  ..........  ..........  ..........  ......Q...  ..........  ..........  ..........  ..........  ..........  ..........  .......... | ..........  ..........  ..........  ..........  ..........  ..........  ..........  ..........  ..........  ..........  ..........  ..........  .......... | ..........  ..........  ..........  ..........  ..........  ..........  .G........  ..........  ..........  ..........  ..........  ..........  .......... | ..........  ..........  ..........  ..........  ..........  ..........  ..........  ..........  ........Y.  ..........  ..........  ..........  .......... | ..........  ..........  ..........  ..........  ..........  ..........  ..........  ..........  ..........  ..........  ..........  ..........  .......... | ..........  ..........  ..........  ..........  ..........  ..........  ..........  ..........  ..........  ..........  ..........  ..........  .......... | ..........  ..........  ..........  ..........  ..........  ..........  ..........  ..........  ..........  ..........  ..........  ..........  .......... |

| fHbp ID1 wildtype | 150  |  FDKLPEGGRA | 160  |  TYRGTAFGSD | 170  |  DAGGKLTYTI | 180  |  DFAAKQGNGK | 190  |  IEHLKSPELN | 200  |  VDLAAADIKP | 210  |  DGKRHAVISG |
| --- | --- | --- | --- | --- | --- | --- | --- |
| yeast-15  yeast-54  yeast-49  yeast-42  yeast-57  yeast-13  yeast-27  yeast-17  yeast-06  yeast-14  yeast-45  yeast-55  yeast-48 | ..I.......  ..N.......  ..E.......  .Y........  .V........  .G........  ..E...D...  ..........  ..........  ..........  ..........  ..I.......  ...P...... | ..........  ..........  ..........  ..........  ..........  ......L...  ..........  ........G.  ..........  ..........  ..........  ..........  .......... | ..........  ..........  ..........  ..........  ..........  ..........  ..........  ..........  ..........  ..........  ..........  ..........  .......... | ..........  ..........  ..........  ..........  ..........  ..........  ..........  ..........  ..........  ..........  ..........  ..........  .......... | ..........  ..........  ..........  ..........  ..........  ..........  ..........  ..........  ..........  ..........  ..........  ....Q.....  .......... | ..........  ..........  ..........  ..........  ..........  ..........  ..........  ..........  ..........  ..........  ..........  ..........  .........L | ..........  ..........  ..........  ..........  ..........  ..........  ..........  ...C......  ...L......  ...C......  ...C......  ..........  .......... |

| fHbp ID1 wildtype | 220  |  SVLYNQAEKG | 230  |  SYSLGIFGGK | 240  |  AQEVAGSAEV | 250  |  KTVNGIRHIG | 255  |  LAAKQ |  |  |
| --- | --- | --- | --- | --- | --- | --- | --- |
| yeast-15  yeast-54  yeast-49  yeast-42  yeast-57  yeast-13  yeast-27  yeast-17  yeast-06  yeast-14  yeast-45  yeast-55  yeast-48 | ..........  ..........  ..........  ..........  ..........  ..........  ..........  ..........  ..........  ..........  .......... ..........  .......... | ..........  ..........  ..........  ..........  ..........  ..........  ..........  ..........  ..........  ..........  ..........  ..........  .......... | ..........  ..........  ..........  ..........  ..........  ..........  ..........  ..........  ..........  ..........  ..........  ..........  .......... | ..........  ..........  ..........  ..........  ..........  ..........  ..........  ..........  ..........  ..........  ..........  ..........  .......... | .....  .....  .....  .....  .....  .....  .....  .....  .....  .....  .....  .....  ..... |  |  |
