# Supplementary figures and images for: Monoclonal Antibodies to Meningococcal Factor H Binding Protein with Overlapping Epitopes and Discordant Functional Activity
Source: PLoS One. 2012 Mar 26;7(3):e34272. doi: 10.1371/journal.pone.0034272 (PMC3312907; doi:10.1371/journal.pone.0034272)

**Figure S2.**


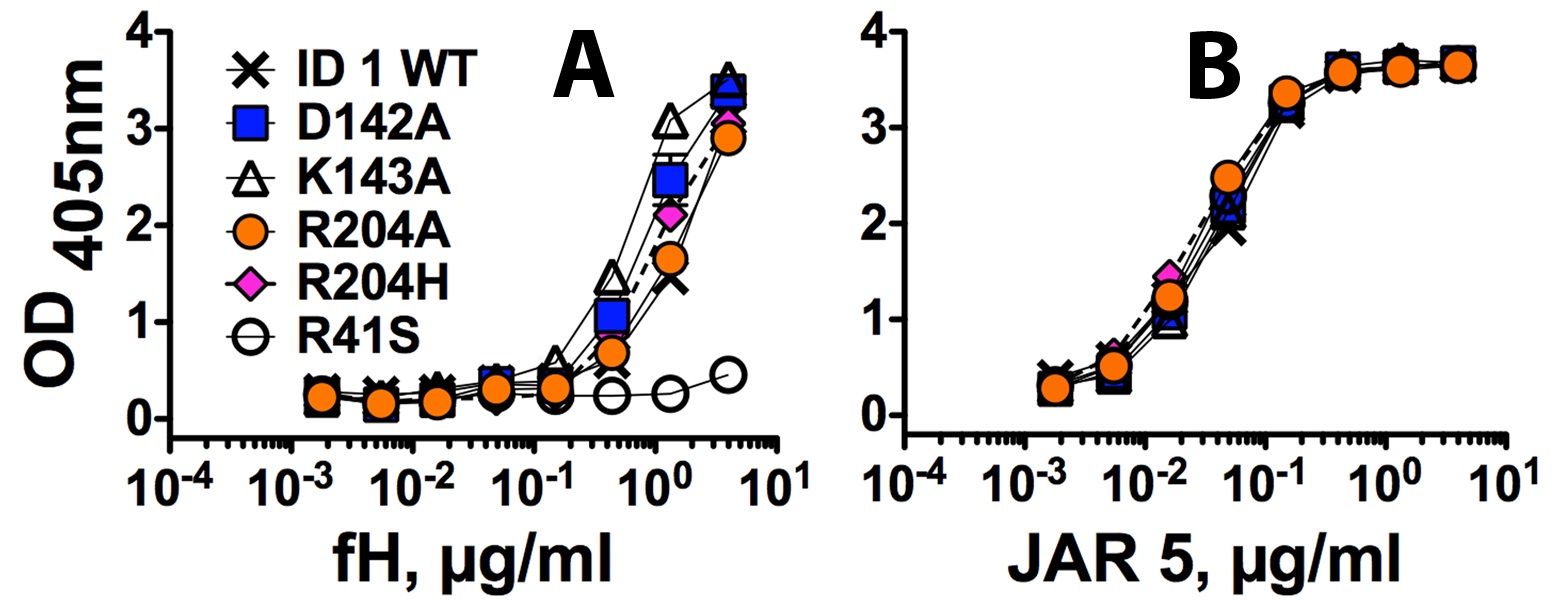

Supplement: Figure S2 — Binding of fH to mutants of fHbp ID 1 as measured by ELISA. Panel A. Binding of fH. The D142A, K433A, R204 and R204H mutations, which affected binding of JAR 1 and/or mAb502, did not affect fH binding. The R41S mutant of fHbp ID (white circles), which was known not to bind fH, served as a negative control [43]. (Panel B. Binding of anti-fHbp mAb, JAR 5. Symbols same as in Panel A. Wells were coated with 2 µg/ml of wild-type or mutant fHbp. (DOC) [file pone.0034272.s002.doc]
